# Comparison of the prognoses of laryngeal preneoplastic lesions based on Ljubljana and World Health Organization classifications

**DOI:** 10.55730/1300-0144.5596

**Published:** 2022-11-04

**Authors:** Ersoy DOĞAN, Cafer BORAN, Mustafa Cüneyt CEVİZCİ, Sülen SARIOĞLU

**Affiliations:** 1 Department of Otorhinolaryngology Head and Neck Surgery, Faculty of Medicine, Dokuz Eylül University, İzmir, Turkey; 2 Department of Pathology, Faculty of Medicine, Dokuz Eylül University, İzmir, Turkey

**Keywords:** Ljubljana classification, preneoplastic, dysplasia, WHO classification, laryngeal precancerous lesions, laryngeal intraepithelial lesions

## Abstract

**Background/aim:**

The aim of this study is to evaluate the prognosis of patients with laryngeal preneoplastic lesions based on Ljubljana classification (LC), Revised LC, World Health Organization Dysplasia System (WHO-DS) 2005 and WHO-DS 2017.

**Materials and methods:**

Patients diagnosed with a laryngeal preneoplastic lesion in our clinic between 2005 and 2018 were included in the study. Biopsy preparations of patients were reexamined by the pathology unit and classified based on LC, Revised LC, WHO-DS 2005, and WHO-DS 2017. Patients with carcinoma were identified during follow-up. The prognosis of preneoplastic lesions was statistically analyzed based on carcinoma development and duration using these four different classifications.

**Results:**

Carcinoma developed in 16 of 142 patients after repeated biopsy. The risk for carcinoma development was found to be more statistically significant in atypical hyperplasia than in squamous cell hyperplasia and basal-parabasal cell hyperplasia according to LC (p: 0.027 and 0.035), no statistically significant difference was observed between squamous and basal-parabasal cell hyperplasia and CIS groups. The risk of carcinoma development was more statistically significant in high-grade squamous intraepithelial lesion (SIL) than in low-grade SIL according to revised LC (p: 0.04); in severe hyperplasia than in other groups according to WHO-DS 2005; and in high-grade dysplasia than in low-grade dysplasia according to WHO-DS 2017 (p: 0.013).

The Cox regression analysis demonstrated that the risk of developing carcinoma statistically increased with age in all classifications, independent of the severity of dysplasia (p < 0.01). According to Cox regression analysis, there was no effect of sex on carcinoma development.

**Conclusion:**

In revised classifications, such as the revised LC and WHO-DS 2017, it is seen that facilitating clinical use is achieved by reducing the number of subgroups by combining the subgroups that do not statistically differ in terms of carcinoma development.

## 1. Introduction

Laryngeal carcinogenesis is not yet fully understood and is defined as a multistage process characterized by cytological changes in the laryngeal squamous epithelium with the progressive contribution of genetic and environmental factors. It was found that carcinoma developed from lesions defined as laryngeal intraepithelial lesions and contain more than one process. Several studies have analyzed the etiological, genetic, and immunological factors that contribute to the development process of these lesions. Despite the extensive research in the field of molecular genetics, reliable molecular markers with diagnostic and prognostic values are still lacking. Traditional light microscopic examinations and findings remain the diagnostic basis on risk factors for laryngeal preneoplastic lesions, such as smoking, alcohol, laryngopharyngeal reflux, human papilloma virus infection, and genetic changes [1]. Pathologists could not reach a consensus for a long time in classifying these lesions using the World Health Organization Dysplasia System (WHO-DS) classification, squamous intraepithelial neoplasia, and Ljubljana classification (LC) [2,3]. The primary goal in making these classifications is to predict the possibility of intraepithelial laryngeal lesion transformation into carcinoma [3,4]. The WHO-DS and Ljubljana classifications are the most common routine methods used to diagnose precancerous laryngeal lesions [5,6]. Due to differences in these three different classification systems, studies have been made on creating a common classification system. Gale et al.’s study made a system using LC and reported that squamous hyperplasia and basal-parabasal hyperplasia have a low potential for carcinoma development and can be combined as a single lesion [7]. Thereafter, according to the revised Ljubljana classification (RLC) made in 2014, three subtypes were defined as low-grade dysplasia, high-grade dysplasia, and carcinoma in situ (CIS). In the new classification published by WHO in 2017, two subtypes, low- and high-grade dysplasia, were developed [8].

Therefore, this study aimed to determine the rate of carcinoma development and its risk factors in patients with laryngeal preneoplastic lesions and to compare the prognosis of preneoplastic laryngeal lesions based on old and new classifications.

## 2. Materials and methods

This retrospective cohort study reviewed the archive materials and file information conducted in Dokuz Eylül University Faculty of Medicine, Department of Otorhinolaryngology, and Department of Pathology. Hospital records of all patients diagnosed with laryngeal preneoplastic lesions and treated and followed up in our clinic between 2005 and 2018 were retrospectively analyzed. The pathology archive materials of these patients were reviewed and reclassified using LC, RLC, WHO-DS 2005, and WHO-DS 2017 classifications.

Patients who applied to our clinic with complaints of voice hoarseness or changes, who underwent direct laryngoscopy and biopsy under general anesthesia, and whose pathology was reported as hyperplasia or dysplasia were included in our study between 2005 and 2018. Age, sex, the severity of dysplasia, number of biopsies, biopsy dates, last control date, control examination findings, development and duration of carcinoma, and treatments received by patients diagnosed with laryngeal preneoplastic lesion were recorded. Statistical analyses were independently performed based on the duration and carcinoma development at least 6 months later. Data were recorded in the data registration form prepared as a Microsoft Excel 2016 file on a personal computer running Windows 10 operating system and then transferred to the Statistical Package for the Social Sciences 22. The rate and time of carcinoma development from dysplastic laryngeal lesions and Kaplan-Meier survival curves were created, and statistical significance was investigated using log-rank and chi-square tests. Significant factors such as age and sex were evaluated using Cox regression analysis (Table 1). p < 0.05 was considered statistically significant. The approval of the Dokuz Eylül University Medical Faculty Non-Invasive Ethics Committee was obtained for this study.

## 3. Results

A total of 169 patients, comprising 141 males (83.4%) and 28 females (16.6%), who underwent direct laryngoscopy and biopsy due to vocal cord lesions between 2005 and 2018 and who were diagnosed with laryngeal preneoplastic lesion after pathological examination were included in this study. The average age of patients was 54.7 years. The average age was 56.7 and 47.6 years for males and females, respectively. In 127 of 169 patients diagnosed with preneoplastic lesions and DL was performed only once, 30 patients received biopsy for the second time, 9 patients for the third time, and 3 patients for the fourth time.

Thirteen patients who never came for control after direct laryngoscopy and biopsy were excluded from statistical analysis. A total of 14 out of 17 patients diagnosed with CIS after the first DL were treated based on the treatment protocols of our clinic. Radiotherapy was applied for 11 of these patients, and surgery (frontolateral laryngectomy, vertical laryngectomy, and type 4 cordectomy) was performed on three of these patients. These 14 patients were also excluded from the statistical analysis due to their treatment. Two patients with CIS who were followed up without treatment were included in the analysis. The average follow-up time and control examination findings of the remaining 142 patients were evaluated based on their last control dates. Accordingly, an average follow-up period of 48 months was obtained postprocedure.

A total of 213 biopsy preparations were reexamined by a pathologist who specialized in head and neck pathology in the Department of Pathology. The first biopsy results of patients were reclassified using LC, RLC, WHO-DS 2005, and WHO-DS 2017. The number of patients by sex, mean ages and mean rates based on the severity of dysplasia is shown in Table 2.

During the follow-up of 142 patients, carcinoma development, carcinoma development time, and the severity of dysplasia based on the first biopsy were evaluated.

Carcinoma developed in 16 patients after repeated biopsy. RT was administered to 15 of 16 patients, and type 4 cordectomy was performed to 1 patient. Total laryngectomy was performed in one patient because of recurrence occurring 1-year post-RT. One patient, who was simultaneously diagnosed with pulmonary carcinoma as a second primary tumor, died. Again in this group, three patients died due to second primary tumors such as prostate, pulmonary, and oropharynx carcinoma during follow-up.

According to Cox regression analysis, the risk of carcinoma development increased statistically as age increased, regardless of the severity of dysplasia (p < 0.01). No effects have been observed on sex in carcinoma development.

The number of patients who developed carcinoma using LC, RLC, WHO-DS 2005, and WHO-DS 2017 classifications; the earliest and latest carcinoma development times; and the rates of carcinoma diagnosis by groups are displayed in Table 3.

**Figure 1:**
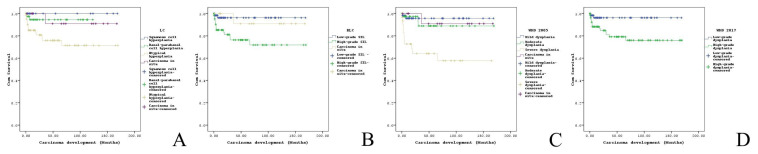
Kaplan-Meier survival curves according to LC (A), RLC (B), WHO-DS 2005 (C) and WHO-DS 2017 (D) classifications (independent of follow-up time). LC: Ljubljana classification; RLC: revised Ljubljana classification; WHO-DS 2005: World Health Organization Dysplasia System 2005; WHO-DS 2017: World Health Organization Dysplasia System 2017.

The risk of carcinoma development was determined to be more statistically significant in atypical hyperplasia in LC than in squamous cell and basal-parabasal hyperplasia (p: 0.027 and 0.035). No statistically significant difference was observed between squamous and basal-parabasal cell hyperplasia and CIS groups (p > 0.05). Statistically significant results were obtained between low- and high-grade squamous intraepithelial lesion (SIL) based on carcinoma development in RLC (p: 0.04). In WHO-DS 2005 and WHO-DS 2017, the risk of carcinoma development was more statistically significant in severe dysplasia than in other groups (p < 0.05) and in high- than low-grade dysplasia (p: 0.013). Kaplan-Meier survival curves according to LC, RLC, WHO-DS 2005 and WHO-DS 2017 classifications are shown in Figures 1A–1D.

To analyze carcinoma development rates depending on the follow-up period, 99 patients who followed up for at least 6 months were evaluated separately. The follow-up period of 43 patients was <6 months.

The number of patients who developed carcinoma based on LC, RLC, WHO-DS 2005, and WHO-DS 2017 classifications, the earliest and latest carcinoma development times, and the rates of carcinoma development by groups are presented in Table 4.

**Figure 2:**
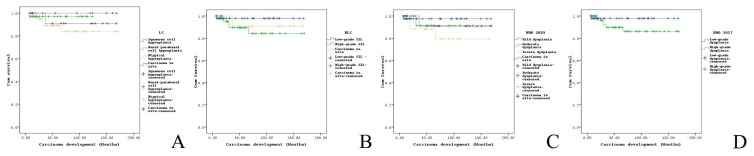
Kaplan-Meier survival curves according to LC (A), RLC (B), WHO-DS 2005 (C) and WHO-DS 2017 (D) classifications (patients with at least 6 months of follow-up). LC: Ljubljana classification; RLC: revised Ljubljana classification; WHO-DS 2005: World Health Organization Dysplasia System 2005; WHO-DS 2017: World Health Organization Dysplasia System 2017.

No statistically significant difference was observed between the severity of dysplasia and carcinoma development among all groups in LC (p > 0.05). Statistically significant results were obtained between low- and high-grade SIL-based on carcinoma development in RLC (p < 0.05). In WHO-DS 2005, severe dysplasia was statistically more risky than mild dysplasia based on carcinoma development (p < 0.05). According to WHO-DS 2017, no statistically significant difference was observed between the groups based on carcinoma development (p > 0.05). Kaplan-Meier survival curves according to LC, RLC, WHO-DS 2005 and WHO-DS 2017 classifications are shown in Figures 2A–2D.

## 4. Discussion

Laryngeal dysplasia is a preneoplastic process with an incidence of 2–10/100,000 individuals [9]. Although it is well-established for >100 years that precancerous lesions of the larynx usually occur before the laryngeal cancer development, the exact progression of such lesions to invasive carcinoma has not yet been fully understood [10,11]. The diagnosis of laryngeal SIL is based on traditional light microscopy, despite the subjectivity of interpretation [2,6].

The rationale of histological rating systems intended for laryngeal SILs should allow a reliable prediction of biological behavior and provide guidance based on the disease treatment and follow-up [7,12]. Preneoplastic changes occur in various lesions and different classification systems have been used to identify such lesions. The aforementioned classifications were based on cellular and epithelial structural changes. The World Health Organization (WHO) and Ljubljana classifications are the most commonly used classification systems in relevant literature [13–15]. Accordingly, the WHO and Ljubljana dysplasia classifications were used in this study. The present study is the first of its kind to investigate the prognosis of preneoplastic lesions by comparing the legacy and revised classifications of both the WHO and Ljubljana.

Lesions, generally considered mild dysplasia, are classified as low-grade dysplasia, whereas lesions, classified as moderate dysplasia, severe dysplasia, and CIS, are classified as high-grade dysplasia. Carcinoma occurs in 40% of high-grade dysplasia lesions, whereas malignant progression occurs in 2% of the low-grade dysplasia cases [16,17]. According to the revised WHO classification of 2017, mild dysplasia, which was included in the previous WHO classification of 2005, was classified as low-grade dysplasia, whereas moderate dysplasia, severe dysplasia, and CIS were classified as high-grade dysplasia. The 5th edition of WHO classification published after our study in 2022 does not present significant changes compared to WHO 2017 [18].

A study conducted by Zhang based on the WHO 2005 classification suggested that mild dysplasia behaved differently as regards the carcinoma development compared with moderate dysplasia, severe dysplasia, and CIS [17]. A metaanalysis by Weller et al. encompassing nine studies with 940 patients who had laryngeal dysplasia reported a total malignant transformation rate of 14%. Mild and moderate dysplasia had a malignant transformation rate of 10.8% compared with 30.4% in severe dysplasia/CIS, and a statistically significant difference was observed between the two groups. The average rate of carcinoma occurrence was 5.8 years [19].

In the present study, based on the WHO 2005 classification, carcinoma occurred in 2 of 69 patients who had mild dysplasia (2%), 3 of 39 patients had moderate dysplasia (7.6%), and 11 of 32 patients had severe dysplasia (34.3%). As patients with severe dysplasia were statistically at a higher risk for carcinoma occurrence compared with other groups (p < 0.05), no statistically significant difference was observed between mild and moderate dysplasia. A review based on the WHO 2017 classification indicated that carcinoma occurred in 2 of 75 patients with low-grade dysplasia (2.6%) and 14 of 67 patients with high-grade dysplasia (20.8%). Furthermore, the risk for carcinoma occurrence in patients with high-grade dysplasia was statistically significantly higher than that in those with low-grade dysplasia (p < 0.05). Based on the above results, no statistically significant difference was observed, although the progression rate of moderate dysplasia to carcinoma was higher than that of mild dysplasia based on percentage. This difference could be attributed to the limited number of patients. Accordingly, we suggest that the medium dysplasia group in the WHO 2005 classification should be compared with severe dysplasia and CIS and investigate the same under the title of high-grade dysplasia as specified in the WHO 2017 classification.

Gale et al. reported a statistically significant difference based on RLC in the carcinoma occurrence between the low- (squamous and basal-parabasal hyperplasia) and high-grade SIL (atypical hyperplasia) groups [2].

In the present study, none of 31 patients with squamous cell hyperplasia developed carcinoma based on LC, whereas two of 45 patients with basal-parabasal cell hyperplasia (4.4%) and 14 of 64 patients with atypical hyperplasia (21.8%) developed carcinoma. A statistically significant difference was observed in carcinoma occurrence among atypical, squamous cell, and basal-parabasal cell hyperplasia. No statistically significant difference was observed in carcinoma occurrence between squamous and basal-parabasal cell hyperplasia. According to RLC, carcinoma occurred in 2 of 75 patients with low-grade SIL (2.6%) and 14 of 65 patients with high-grade SIL (21.5%). The risk for developing carcinoma in patients with high-grade SIL was statistically significantly higher than in those with low-grade SIL (p < 0.05). One patient with basal-parabasal hyperplasia based on LC was included in the high-grade SIL group based on RLC. These findings are suggestive of the fact that the classification of squamous and basal-parabasal cell hyperplasia in LC in the low-grade SIL group, unlike the RLC, was aimed at decreasing the number of subgroups to facilitate the use of the classification system.

Gallo et al., in the study with 259 patients, reported a significant relationship between the severity of dysplasia and duration of carcinoma development in 19 patients (17 men and 2 women) who developed carcinoma during follow-up [4]. Spielman et al. suggested that patients with mild and moderate dysplasia who were followed up for 3 years and did not develop carcinoma could be excluded from further follow-up [20]. Similarly, Plch et al. reported no carcinoma development in patients with mild and moderate dysplasia upon a 20-year follow-up [21]. Nevertheless, Theodosiou et al. found that four patients with mild and moderate dysplasia developed carcinoma after a >5-year follow-up [22]. In the present study, 2 of 69 patients with mild dysplasia and 3 of 39 patients with moderate dysplasia based on the WHO 2005 classification developed carcinoma during the follow-up period. The longest duration of carcinoma development was 30 months in a patient with moderate dysplasia. The results of our study are similar to that of Spielman et al.’s and suggest that the risk of developing carcinoma decreases after 3 years in patients with mild and moderate dysplasia. Furthermore, 11 of 32 patients with severe dysplasia developed carcinomas, and the longest duration of carcinoma development was 66 months. These findings suggest that patients with severe dysplasia should be followed up for >5 years.

Theodosiou et al. suggested that a minimum of 6 months should pass for the transformation of dysplasia into carcinoma [22]. Another study reported that a more advanced lesion must have been likely skipped during the first biopsy in patients with carcinoma detected in <6 months [17].

In the present study, patients followed up for at least 6 months were further analyzed based on carcinoma development to investigate the relationship between carcinoma development and duration. Accordingly, results indicated that 16 patients developed carcinoma regardless of the duration, whereas 6 patients developed carcinoma after >6 months of follow-up. Carcinoma occurrence in 10 of 16 patients within the first 6 months may suggest that a more advanced lesion might have been skipped during the first biopsy or that biopsy might have been taken from an area of the lesion with a lower dysplasia grade.

Most centers consider that patients previously diagnosed with CIS would develop carcinoma in a short time and at a high rate. Accordingly, as surgery or radiotherapy is considered for patients with CIS, others with dysplasia are followed up without treatment [17]. In patients with CIS, radiotherapy is the appropriate option when complete excision of the lesion is not possible. A metaanalysis by Sadri et al. reported that radiotherapy provided a significantly higher rate of local control compared to surgical treatments because of the wider therapeutic domain associated with the procedure for severe dysplastic lesions and CIS [15]. Conversely, radiotherapy is never used to treat high-grade SIL in the Slovenian group and is reserved for more advanced diseases, including CIS and invasive squamous cell carcinoma [2]. Patients diagnosed with CIS are treated in our clinic. A review of study data showed that 11 of 17 patients diagnosed with CIS after the first DL underwent radiotherapy, 3 underwent surgery (frontolateral laryngectomy, vertical laryngectomy, and type 4 cordectomy), and 2 were followed up without treatment. Furthermore, 14 treated patients and 1 patient without follow-up were excluded from the statistical analysis for carcinoma development. Only two patients who followed up without treatment were classified in the CIS group and did not develop carcinoma. Owing to the limited number of patients, adequate statistical analysis and prognostic interpretation of lesions diagnosed with CIS and followed up without treatment based on LS, RELS, and WHO 2005 classifications were impossible. The moderate dysplasia, severe dysplasia, and CIS groups specified in the WHO 2005 classification were combined and categorized under the high-grade dysplasia group based on the WHO 2017 classification. Accordingly, some patients who were previously treated for CIS could be followed up without treatment because they are now classified in the high-grade dysplasia group based on the new system. Further multicenter studies with prolonged follow-up periods are required to evaluate the prognosis of lesions in the high-grade dysplasia group based on the WHO 2017 classification.

## 5. Conclusion

Different classifications used to histologically rate the laryngeal intraepithelial lesions aimed to provide a reliable prediction of biological behaviors and to guide the disease treatment and follow-up. Classifications are updated and revised forms are published periodically in order to facilitate their clinical use. In both revised LC and WHO-DS 2017, it is seen that facilitating clinical use is achieved by reducing the number of subgroups of LC and WHO-DS 2005 by combining the subgroups that do not statistically differ in terms of carcinoma development. However, since the number and histological nomenclature of the subgroups of LC and WHO-DS are different, statistical comparisons cannot be made between these two classifications in terms of carcinoma development. Revised LC should be reduced into two subgroups as low grade SIL and high grade SIL by combining the high grade SIL and CIS that do not statistically differ in terms of carcinoma development.

## Figures and Tables

**Table 1:** Cox regression models with confounding factors and four classification.

	Exp (B) (OR)	95.0% CI for Exp (B) (OR)		p value
		Lower	Upper	
**Model 1**				
Sex	0.00	0.00		0.977
Age	1.11	1.05	1.17	<0.01
LC	0.98	0.55	1.74	0.934
**Model 2**				
Sex	295391.33	0.00		0.976
Age	1.12	1.06	1.18	< 0.01
RLC	0.77	0.37	1.62	0.49
**Model 3**				
Sex	279958.27	0.00		0.977
Age	1.11	1.05	1.17	< 0.01
WHO-DS 2005	1.03	0.63	1.67	0.907
**Model 4**				
Sex	238024.1	0.00		0.977
Age	1.1	1.04	1.16	< 0.01
WHO-DS 2017	2.09	0.46	9.57	0.343

OR: odds ratio; LC: Ljubljana classification (LC); RLC: revised Ljubljana classification; WHO-DS 2005: World Health Organization Dysplasia System 2005; WHO-DS 2017: World Health Organization Dysplasia System 2017.

**Table 2:** Distribution of patients by LC, RLC, WHO-DS 2005, and WHO-DS 2017.

	Number (female/male)	Rate (%)	Average age
**LC**			
Squamous cell hyperplasia	39 (11/28)	23.1	48.3
Basal-parabasal cell hyperplasia	46 (9/37)	27.2	50.6
Atypical hyperplasia	67 (6/61)	39.6	59.3
CIS	17 (2/15)	10.1	62
**RLC**			
Low-grade SIL	84 (20/64)	49.7	54.9
High-grade SIL	68 (6/62)	40.2	59.2
CIS	17 (2/15)	10.1	62
**WHO-DS 2005**			
Mild dysplasia	78 (19/59)	46.2	49.1
Moderate dysplasia	40 (5/35)	23.7	45.3
Severe dysplasia	34 (2/32)	20.1	59.8
CIS	17 (2/15)	10.1	62
**WHO-DS 2017**			
Low-grade dysplasia	84 (20/64)	49.7	47.7
High-grade dysplasia	85 (8/77)	50.3	56.8

CIS: carcinoma in situ; SIL: squamous intraepithelial lesion; LC: Ljubljana classification (LC); RLC: revised Ljubljana classification; WHO-DS 2005: World Health Organization Dysplasia System 2005; WHO-DS 2017: World Health Organization Dysplasia System 2017.

**Table 3:** Distribution of all patients diagnosed with carcinoma in their follow-up based on LS, RELS, WHO-DS 2005 and 2017 *.

	Number of patients diagnosed with carcinoma/total patients	Average duration of carcinoma diagnosis, earliest-latest (months)	Carcinoma development rate (%)
**LC**			
Squamous cell hyperplasia	0 / 31	-	-
Basal-parabasal cell hyperplasia	2 / 45	3.9 (1.1–6.8)	4.44
Atypical hyperplasia	14 / 64	12.9 (0.37–66.1)	21.8
CIS	0 / 2	-	-
**RLC**			
Low-grade SIL	2 / 75	3.9 (1.1–6.8)	2.6
High-grade SIL	14 / 65	12.9 (0.37–66.1)	21.5
CIS	0 / 2	-	-
**WHO-DS 2005**			
Mild dysplasia	2 / 69	4.57 (1.17–6.80)	2.89
Moderate dysplasia	3 / 39	13.62 (0.63–30.03)	7.69
Severe dysplasia	11 / 32	64.7 (0.37–66.1)	34.3
CIS	0/2	-	-
**WHO-DS 2017**			
Low-grade dysplasia	2 / 75	4.57 (1.17–6.80)	2.66
High-grade dysplasia	14 / 67	12.9 (0.37–66.1)	20.8

* Data independent of follow-up time.

CIS: carcinoma in situ; SIL: squamous intraepithelial lesion; LC: Ljubljana classification; RLC: revised Ljubljana classification; WHO-DS 2005: World Health Organization Dysplasia System 2005; WHO-DS 2017: World Health Organization Dysplasia System 2017.

**Table 4:** Distribution of patients who developed carcinoma in their follow-up based on LC, RLC, WHO-DS 2005, and WHO-DS 2017 *.

	Number of patients diagnosed with carcinoma/total patients	Average duration of carcinoma diagnosis, earliest-latest (months)	Carcinoma development rate (%)
**LC**			
Squamous cell hyperplasia	0/15	-	-
Basal-parabasal cell hyperplasia	1/35	6.8	2.85
Atypical hyperplasia	5/48	32.66 (18.03–66.1)	10.41
CIS	0/1	-	-
**RLC**			
Low-grade SIL	1/49	6.8	2.08
High-grade SIL	5/49	32.66 (18.03–66.1)	10.20
CIS	0/1	-	-
**WHO-DS 2005**			
Mild dysplasia	1/43	6.8	2.38
Moderate dysplasia	2/34	29.91 (29.8–30.03)	5.88
Severe dysplasia	3/21	34.41 (18.03–66.1)	14.28
CIS	0/1	-	-
**WHO-DS 2017**			
Low-grade dysplasia	1/49	6.8	2.08
High-grade dysplasia	5/50	32.66 (18.03–66.1)	10

* Data of patients with at least 6 months of follow-up.

CIS: carcinoma in situ; SIL: squamous intraepithelial lesion; LC: Ljubljana classification; RLC: revised Ljubljana classification; WHO-DS 2005: World Health Organization Dysplasia System 2005; WHO-DS 2017: World Health Organization Dysplasia System 2017.

## References

[1] Thompson LDR Laryngeal dysplasia, squamous cell carcinoma, and variants Surgical Pathology Clinics 2017 10 1 15 33 10.1016/j.path.2016.10.003 28153131

[2] Gale N Blagus R El-Mofty SK Helliwell T Prasad ML Evaluation of a new grading system for laryngeal squamous intraepithelial lesions-a proposed unified classification Histopathology 2014 65 4 456 464 10.1111/his.12427 24689850

[3] Hellquist H Ferlito A Makitie AA Thompson LDR Bishop JA Developing classifications of laryngeal dysplasia: The historical basis Advances in Therapy 2020 37 6 2667 2677 10.1007/s12325-020-01348-4 32329013 PMC7467449

[4] Gallo A de Vincentiis M Della Rocca C Moi R Simonelli M Evolution of precancerous laryngeal lesions: A clinicopathologic study with long-term follow-up on 259 patients Head & Neck 2001 23 1 42 47 10.1002/1097-0347(200101)23:1<42:AID-HED7>3.0.CO;2-1 11150070

[5] Sengiz S Pabuççuoğlu U Sarioğlu S Immunohistological comparison of the World Health Organization (WHO) and Ljubljana classifications on the grading of preneoplastic lesions of the larynx Pathology Research and Practice 2004 200 3 181 188 10.1016/j.prp.2003.11.002 15200269

[6] Odell E Eckel HE Simo R Quer M Paleri V European Laryngological Society position on laryngeal dysplasia Part I: aetiology and pathological classification European Archives of Otorhinolaryngology 2021 278 6 1717 1722 10.1007/s00405-020-06403-y 33051798 PMC8131293

[7] Gale N Michaels L Luzar B Poljak M Zidar N Current review on squamous intraepithelial lesions of the larynx Histopathology 2009 54 6 639 656 10.1111/j.1365-2559.2008.03111.x 18752537

[8] Gale N Poljak M Zidar N Update from the 4th edition of the World Health Organization of head and neck tumours: What is new in the 2017 WHO blue book for tumours of the hypopharynx, larynx, trachea, and parapharyngeal space Head and Neck Pathology 2017 11 1 23 32 10.1007/s12105-017-0788-z 28247231 PMC5340729

[9] Bouquot JE Gnepp DR Laryngeal precancer: A review of the literature, commentary, and comparison with oral leukoplakia Head & Neck 1991 13 6 488 497 10.1002/hed.2880130604 1791144

[10] Lentsch EJ Myers JN New trends in the etiology, diagnosis, and management of laryngeal dysplasia Current Opinion in Otolaryngology Head and Neck Surgery 2001 9 2 74 78 10.1097/00020840-200104000-00003

[11] Gale N Cardesa A Hernandez-Prera JC Slootweg PJ Wenig BM Laryngeal dysplasia: persisting dilemmas, disagreements and unsolved problems-A short review Head and Neck Pathology 2020 14 4 1046 1051 10.1007/s12105-020-01149-9 32141027 PMC7669915

[12] Fleskens SA Bergshoeff VE Voogd AC van Velthuysen ML Bot FJ Interobserver variability of laryngeal mucosal premalignant lesions: A histopathological evaluation Modern Pathology 2011 24 7 892 898 10.1038/modpathol.2011.50 21499237

[13] Sarioglu S Cakalagaoglu F Elagoz S Han U Etit D Inter-observer agreement in laryngeal pre-neoplastic lesions Head and Neck Pathology 2010 4 276 280 10.1007/s12105-010-0208-0 20857246 PMC2996497

[14] Hellquist H Cardesa A Gale N Kambic V Michaels L Criteria for grading in the Ljubljana classification of epithelial hyperplastic laryngeal lesions. A study by members of the Working Group on Epithelial Hyperplastic Laryngeal Lesions of the European Society of Pathology Histopathology 1999 34 3 226 233 10.1046/j.1365-2559.1999.00581.x 10217563

[15] Sadri M McMahon J Parker A Management of laryngeal dysplasia: A review European Archives of Otorhinolaryngology 2006 263 9 843 852 10.1007/s00405-006-0078-y 16823559

[16] Karataylı-Ozgursoy S Pacheco-Lopez P Hillel AT Best SR Bishop JA Laryngeal dysplasia, demographics, and treatment: A single-institution, 20-year review JAMA Otolaryngol - Head Neck Surg 2015 141 4 313 318 10.1001/jamaoto.2014.3736 25654369

[17] Zhang HK Liu HG Is severe dysplasia the same lesion as carcinoma in situ? 10-year follow-up of laryngeal precancerous lesions Acta Oto-Laryngologica 2012 132 3 325 328 10.3109/00016489.2011.642812 22229875

[18] Zidar N Gale N Update from the 5th Edition of the World Health Organization Classification of Head and Neck Tumors: Hypopharynx, Larynx, Trachea and Parapharyngeal Space Head and Neck Pathology 2022 16 1 31 39 10.1007/s12105-021-01405-6 35312977 PMC9018940

[19] Weller MD Nankivell PC McConkey C Paleri V Mehanna HM The risk and interval to malignancy of patients with laryngeal dysplasia; a systematic review of case series and meta-analysis Clinical Otolaryngology 2010 35 5 364 372 10.1111/j.1749-4486.2010.02181.x 21108746

[20] Spielmann PM Palmer T McClymont L 15-year review of laryngeal and oral dysplasias and progression to invasive carcinoma European Archives of Otorhinolaryngology 2010 267 3 423 427 10.1007/s00405-009-1013-9 19543904

[21] Plch J Pár I Navrátilová I Bláhová M Zavadil M Long term follow-up study of laryngeal precancer Auris Nasus Larynx 1998 25 4 407 412 10.1016/s0385-8146(98)00041-8 9853665

[22] Theodosiou MG Yiotakis J Dikoglou C Lazaris AC Athanasiadis-Sismanis A Laryngeal dysplasia: A long-term follow-up study Journal of B.U.O.N 2013 18 3 683 688 24065483

